# Role of hydrogen sulfide in subarachnoid hemorrhage

**DOI:** 10.1111/cns.13828

**Published:** 2022-03-22

**Authors:** Dengfeng Lu, Lingling Wang, Guangjie Liu, Shixin Wang, Yi Wang, Yu Wu, Jing Wang, Xiaoou Sun

**Affiliations:** ^1^ Department of Neurosurgery & Brain and Nerve Research Laboratory The First Affiliated Hospital of Soochow University Suzhou Jiangsu Province China

**Keywords:** apoptosis, cerebral edema, hydrogen sulfide, inflammation, neuroprotection, oxidative stress, subarachnoid hemorrhage, vasospasm

## Abstract

Subarachnoid hemorrhage (SAH) is a common acute and severe disease worldwide, which imposes a heavy burden on families and society. However, the current therapeutic strategies for SAH are unsatisfactory. Hydrogen sulfide (H_2_S), as the third gas signaling molecule after carbon monoxide and nitric oxide, has been widely studied recently. There is growing evidence that H_2_S has a promising future in the treatment of central nervous system diseases. In this review, we focus on the effects of H_2_S in experimental SAH and elucidate the underlying mechanisms. We demonstrate that H_2_S has neuroprotective effects and significantly reduces secondary damage caused by SAH via antioxidant, antiinflammatory, and antiapoptosis mechanisms, and by alleviating cerebral edema and vasospasm. Based on these findings, we believe that H_2_S has great potential in the treatment of SAH and warrants further study to promote its early clinical application.

## Graphical abstract

Hydrogen sulfide was found to exert neuroprotective effects postexperimental subarachnoid hemorrhage (SAH) and could significantly reduce secondary damage caused by SAH via antioxidant, antiinflammatory effect, antiapoptosis, and alleviating cerebral edema and cerebral vasospasm.

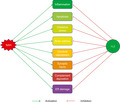

## 1 INTRODUCTION

Although subarachnoid hemorrhage (SAH) accounts for only 5% of stroke incidence, its mortality rate is as high as 44%, bringing a heavy economic burden to society and families.^1^, ^2^ Moreover, survivors of SAH are often accompanied by multiple neurological deficits that severely affect patients’ ability to work and their quality of life.^2^, ^3^ Existing treatments for SAH remain unsatisfactory. Recently, hydrogen sulfide (H_2_S), the third gaseous signaling molecule, has been found to play an important role in physiological processes.^4^, ^5^, ^6^, ^7^ However, the role of H_2_S in SAH has been rarely reported. It has been hypothesized that H_2_S may have a neuroprotective effect in brain injury caused by SAH,^8^ which was later confirmed by a series of preclinical studies. Here, we summarize the role of H_2_S therapy and its potential mechanism in SAH to provide a novel idea for treating SAH.

## 2 AN OVERVIEW OF SAH

Stroke is the second leading cause of death and the third leading cause of disability worldwide. Spontaneous SAH accounts for approximately 5% of strokes. Although less common than ischemic stroke and intracerebral hemorrhage, the high mortality rate of SAH imposes a heavy burden on society and families.^9^ Approximately 85% of spontaneous SAH cases are caused by ruptured aneurysms, while other causes include trauma, cerebrovascular malformation, moyamoya disease, amyloid vascular disease, cerebral venous thrombosis, and pituitary stroke.^2^, ^10^ Sudden onset of severe headache is the main symptom of SAH, while some patients may also experience nausea, vomiting, seizures, transient or persistent loss of consciousness, or focal neurological deficits.^2^, ^3^, ^10^, ^11^ Head computed tomography (CT) scan and lumbar puncture are the two main methods to diagnose SAH.^1^, ^3^, ^12^ Once a diagnosis of SAH is made, vascular imaging should be performed to determine the cause of the onset of SAH (usually a ruptured aneurysm).^3^, ^12^ Common treatment options for aneurysms in patients with SAH include endovascular intervention and surgical clipping of aneurysms.^12^ Despite great advances in the treatment of patients with aneurysmal SAH, the mortality is still high at 32%–67%, and one‐third of SAH survivors remain dependent on others.^10^

## 3 MECHANISM OF SAH

Brain injury caused by SAH consists of two stages: early brain injury (EBI) and a delayed brain injury stage, often referred to as delayed cerebral ischemia (DCI).^2^ EBI, which occurs within 72 h of SAH onset, is considered to be caused by transient global ischemia, toxic effects of subarachnoid blood, and direct destruction of brain tissue by hemorrhage, and it is a major factor leading to poor prognosis.^2^, ^13^, ^14^ Although the pathogenesis of EBI after SAH has not been fully elucidated, studies suggest that it may be associated with abnormal inflammatory responses, apoptosis, oxidative stress, blood‐brain barrier (BBB) disruption, endoplasmic reticulum (ER) stress, cerebral edema, and cerebral vasospasm.^15^, ^16^, ^17^, ^18^, ^19^, ^20^ DCI refers to a complex of reaction that occurs following SAH. DCI is caused by various mechanisms, including angiographic cerebral vasospasm, microvascular spasm, microthrombosis, cortical spreading depolarization, failure of cerebral autoregulation, and inflammatory responses.^17^, ^21^ Delayed neurological deterioration due to DCI occurs in approximately one‐third of patients within 3–14 days after SAH.^2^ Current therapeutic effects are unsatisfactory, and further studies on these mechanisms are urgently needed to better solve this problem.

## 4 PRODUCTION, CATABOLISM, AND STORAGE OF H_2_S IN VIVO

**FIGURE 1:**
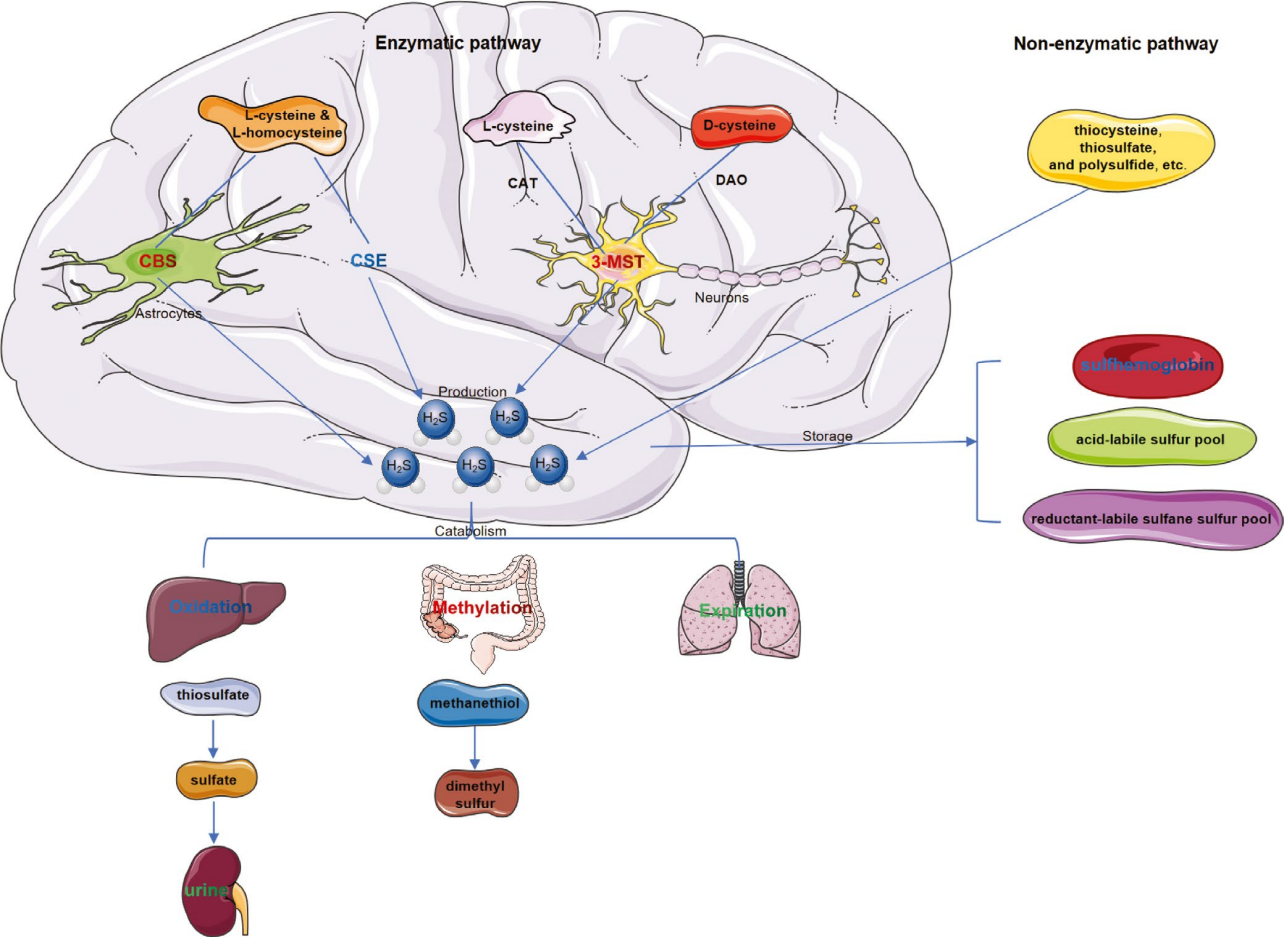
Current understanding of production, catabolism, and storage of H_2_S in vivo, especially in the brain. CAT, cysteine aminotransferase; CBS, cystathionine‐β‐synthetase; CSE, cystathionine‐γ‐lyase; DAO, D‐amino acid oxidase; MST, mercaptopyruvate sulfurtransferase

Hydrogen sulfide is a colorless, flammable, water‐ and lipid‐soluble gas with a distinctive smell of rotten eggs. For decades, H_2_S was primarily concerned as a toxic gas and an environmental hazard, but it is also produced in mammals, including humans, and it can be detected in large quantities.^22^ Owing to its good water and lipid solubility, H_2_S easily passes through the plasma membrane.^23^ H_2_S is currently recognized as the third gaseous transmitter following nitric oxide (NO) and carbon monoxide (CO). As one of the most important signal molecules, H_2_S participates in many biological processes.^5^, ^22^ Figure 1 briefly illustrates our current understanding of H_2_S production, catabolism, and storage in vivo, especially in the brain.

## 5 PRODUCTION OF H_2_S

In mammalian cells, endogenous H_2_S is generated via enzymatic and nonenzymatic pathways. The enzymatic pathway of H_2_S synthesis involves three key enzymes: cystathionine‐β‐synthetase (CBS), cystathionine‐γ‐lyase (CSE), and 3‐mercaptopyruvate sulfurtransferase (3‐MST).^24^ Specifically, the main H_2_S‐producing enzymes in the brain are CBS and 3‐MST.^25^ CBS mainly exists in astrocytes, while 3‐MST mainly exists in neurons.^26^ In the cytoplasm of glial cells, as pyridoxal 5′‐phosphate (PLP)‐dependent enzymes, CBS synthesizes H_2_S by catalyzing l‐cysteine and l‐homocysteine.^27^, ^28^ PLP‐independent 3‐MST exists in both the cytoplasm and mitochondria of neurons.^24^ In the presence of cysteine aminotransferase (CAT), 3‐MST generates H_2_S from l‐cysteine. In the presence of D‐amino acid oxidase (DAO), 3‐MST can also catalyze H_2_S production with d‐cysteine as substrate.^23^ In addition to the expression and distribution of the enzymes above, the production of endogenous H_2_S depends on the availability of substrates and enzyme activity, which may be affected by many properties of the intracellular environment, such as the redox state.^6^ Some sulfur‐containing substances, such as thiocysteine, thiosulfate, and polysulfide, can also generate endogenous H_2_S through nonenzymatic pathways, but this accounts for only a small portion of H_2_S production.^29^

## 6 CATABOLISM OF H_2_S

Less is known about the catabolism of H_2_S compared with the synthesis. At present, catabolic routes of H_2_S mainly include oxidation, methylation, and expiration.^30^ The oxidation of H_2_S occurs mainly in the liver.^31^ Hydrogen sulfide is oxidized to form thiosulfate directly or indirectly in mitochondria.^32^, ^33^, ^34^, ^35^, ^36^ The sulfide‐detoxifying enzymes catalyze the conversion of thiosulfates to sulfates.^37^, ^38^ As a result, most H_2_S in vivo is excreted in the urine as sulfate. In one study, sulfide oxidation due to increased sulfide quinone oxidoreductase (SQR, one of the key enzymes during H_2_S oxidation) activity was observed in the kidney, heart, and liver when administered with exogenous H_2_S, but not in brain tissue, indicating a defect of sulfide oxidation in brain tissue.^25^ Since free H_2_S exists at a low concentration in the blood and decays rapidly, it is less likely to be transported to the liver for removal. Whether H_2_S in the brain is catabolized in other ways, or it is transported to the liver or kidney in other forms and catabolized, is still confusing.

Unlike oxidation, methylation of H_2_S occurs mainly in the cytoplasm. H_2_S is first methylated to methanethiol, which is further methylated to nontoxic dimethyl sulfur by thiol S‐methyltransferase.^39^ Sulfide methylation in the colon mucosa is approximately 10,000 times slower than oxidation of sulfide.^38^ Therefore, the methylation pathway of H_2_S catabolism accounts for a small proportion under physiological conditions.

In addition to oxidation and methylation pathways, H_2_S can be exhaled directly through the lungs. H_2_S can be detected in expired air when exogenous sulfur‐containing substances, such as sodium sulfide (Na_2_S) are given or when it is overproduced in pathological conditions, such as septic shock, hemorrhagic shock, and chronic obstructive pulmonary disease.^40^, ^41^, ^42^, ^43^ However, the amount of H_2_S in exhaled gas under physiological conditions has not been accurately reported, probably because the amount is too small to be detected. Therefore, exhalation may serve as a potential pathway for H_2_S expelling when the first two pathways fail to compensate.

## 7 STORAGE OF H_2_S

Hydrogen sulfide mainly exists in the form of gaseous molecules (H_2_S) or sodium bisulfide (NaHS). The two can be converted into each other and maintain a dynamic balance at a ratio of 1:2.^44^ H_2_S can bind to hemoglobin to form sulfhemoglobin, which may be one of the storage forms of H_2_S in vivo.^30^ In addition, the recognized form of H_2_S storage in the body is the acid‐labile sulfur pool such as iron‐sulfur cluster‐containing proteins, and reductant‐labile sulfane sulfur pool, which include hydrodisulfides/persulfides.^45^, ^46^

## 8 ROLE OF H_2_S IN THE CENTRAL NERVOUS SYSTEM

Many reviews have reported the role of H_2_S in the central nervous system (CNS) and its possible mechanisms; thus, we provide only a brief overview below.

### 8.1 Regulation of intracellular signaling pathways

Hydrogen sulfide may regulate long‐term potentiation (LTP) induction/potentiation by activating the cyclic adenosine monophosphate/protein kinase A (cAMP/PKA) pathway and/or receptor tyrosine kinases (RTK), and it plays an important role in neural transmission involved in learning and memory.^26^, ^47^, ^48^ Furthermore, H_2_S is essential for maintaining a balance between oxidation and antioxidant activity in vivo and may play a neuroprotective role by promoting the production of glutathione (GSH, an important reductant in the brain) through different mechanisms to antagonize neuroinflammation and oxidative stress.^26^, ^47^, ^49^, ^50^

### 8.2 Regulation of ion channels

H_2_S could promote an increase in Ca^2+^ concentration in neurons, astrocytes, and even microglia by regulating calcium channels in the plasma membrane. Besides, physiological concentrations of H_2_S may also mobilize Ca^2+^ reservoirs in different cells. Changes in calcium concentration further mediate the regulation of various physiological processes, including neuronal morphogenesis and development, neurotransmitter release, synaptic plasticity, and gene transcription.^47^, ^48^, ^51^ Moreover, H_2_S could result in the current inhibition of several inwardly rectifying potassium (Kir) channels, which results from changes in channel‐gating kinetics. Kir channels establish and regulate the resting membrane potential of excitable cells in the heart, brain, and other peripheral tissues.^52^ In addition, H_2_S can also activate Cl^−^ channels, thus affecting regulation on excitability.

### 8.3 Regulation of amino acid neurotransmitters

Gamma‐aminobutyric acid (GABA) is the major inhibitory transmitter in the CNS of mammals, and the loss of GABAergic inhibition could lead to seizures and hyperexcitability of neurons. H_2_S was reported to promote the amelioration of hippocampal damage induced by recurrent febrile seizures via reversing the loss of GABA receptors. Similarly, H_2_S may play a role in excitatory diseases such as epilepsy by modulating the inhibitory neurotransmitter GABA.^47^ However, H_2_S enhances N‐methyl‐D‐aspartate (NMDA) receptor signaling by activating the cAMP/PKA pathway, thereby stimulating LTP. NMDA receptor is the main receptor of glutamate, which is an important excitatory neurotransmitter in the brain.^53^ Moreover, H_2_S may also regulate neuronal survival/death by regulating the opening of NMDA receptor channels.^47^

In addition, it was reported that H_2_S plays a protective role by antiapoptosis by regulating nuclear translocation of nuclear factor kappa B (NF‐κB) and by stabilizing membrane potentials via regulating ER stress and activating K^+^ channel and Cl^−^ channel.^49^ In conclusion, H_2_S participates in several complex physiological and pathological processes, which lays a foundation for its role in the development of many CNS diseases.

## 9 APPLICATION OF H_2_S IN CNS DISEASES

Traumatic brain injury (TBI) is defined as alterations in brain structure and/or function caused by external forces.^54^ One study in 2003 reported that endogenous H_2_S production was reduced in the cerebral cortex and hippocampus of TBI mice, and intraperitoneal injection of NaHS reduced the volume of TBI‐induced injury.^55^ In the same year, another study confirmed the protective effect of H_2_S on TBI rats. After exogenous H_2_S treatment, the neural function of rats was significantly improved, the activity of endogenous antioxidant enzymes was increased, the level of oxidative products was decreased, and the BBB disruption and brain edema were alleviated. The underlying mechanism could be related to the activation of mitochondrial adenosine triphosphate‐sensitive potassium channels and the reduction in oxidative stress.^56^ Moreover, another study on TBI mice demonstrated that the protective effect of H_2_S in TBI may be associated with the regulation of apoptosis and autophagy.^57^ In 2020, a review by Zhang et al.^46^ further summarized the potential role of H_2_S in TBI. Their work suggested that H_2_S could reduce secondary brain injury after TBI via antioxidation and antiinflammatory effects, and by regulating cell death signaling and alleviating cerebral vasospasm and brain edema.

Ischemic stroke, which accounts for approximately 80% of all strokes, occurs when blood flow to the brain tissue is occluded.^58^ Exogenous H_2_S treatment reduces brain injury and cerebral edema after ischemia in a dose‐dependent manner, possibly by blocking programmed cell death.^59^ Besides, another study in a rat model of global cerebral ischemia‐reperfusion (I/R) also suggested that H_2_S may have a protective effect against severe brain injury induced by global I/R by inhibiting oxidative stress, the inflammatory response, and apoptosis.^60^ In 2016, research in rats and mice by Shi et al. suggested that Na_2_S reduced infarct size, improved cerebral energy metabolism after cerebral global ischemia, and prolonged survival time of animals. Moreover, increased cerebral blood flow and decreased cerebrovascular resistance, blood viscosity, and thrombogenesis were also observed in animals treated with Na_2_S. In cultured neurons, Na_2_S increased cell viability and decreased cell apoptosis induced by oxygen‐glucose deprivation.^61^ In addition, Ren et al. suggested that lower rather than higher concentrations of exogenous H_2_S may offer protection against the neuronal injury induced by I/R.^62^ There does not seem to be a clear answer to the question: “Hydrogen sulfide in stroke: Protective or harmful?” Indeed, the available evidence suggests that the presence of H_2_S in ischemic regions may be either harmful or protective, depending on the concentration.^63^, ^64^

Intracerebral hemorrhage (ICH) is another type of hemorrhagic stroke. In 2020, Zhang et al.^65^ found that endogenous H_2_S production was decreased after ICH, and exogenous H_2_S treatment may alleviate secondary injury induced by ICH through antiinflammatory and antioxidant mechanisms, and by the regulation of autophagy and neuronal death and the alleviation of cerebral edema.

Neurodegenerative diseases are a group of central and peripheral nervous system diseases characterized by chronic neurological dysfunction and neuronal loss, among which Alzheimer's disease (AD) and Parkinson's disease (PD) are the most common.^66^ There is evidence that H_2_S exerts a protective effect in both in vivo and in vitro AD models by interfering with amyloid precursor protein (APP) metabolism, and by mediating antioxidant, antiinflammatory, and antiapoptosis effects.^48^, ^50^, ^67^ Similarly, H_2_S alleviates neuronal degeneration, apoptosis, and inflammatory response in PD animals, suggesting a neuroprotective role against PD.^48^, ^50^ In summary, existing evidence suggests that H_2_S has a neuroprotective effect in various CNS diseases, which makes it reasonable to speculate that brain injury caused by SAH may also benefit from the administration of this gas.

## 10 CHANGES IN ENDOGENOUS H_2_S AND H_2_S‐PRODUCING ENZYMES IN SAH

**FIGURE 2:**
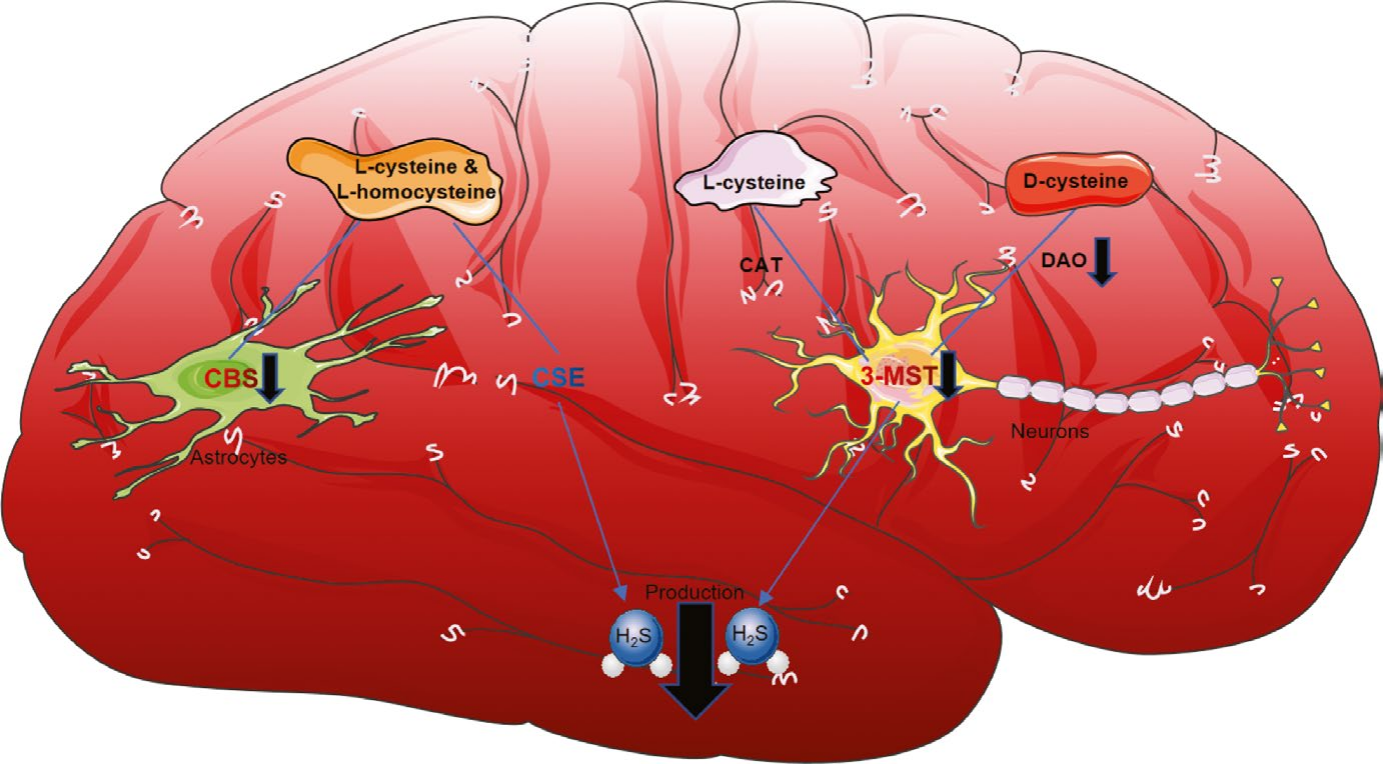
Current understanding of the metabolism of H_2_S and how it changes after SAH. CAT, cysteine aminotransferase; CBS, cystathionine‐β‐synthetase; CSE, cystathionine‐γ‐lyase; DAO, D‐amino acid oxidase; MST, mercaptopyruvate sulfurtransferase

So far, several studies have reported changes in endogenous H_2_S and its producing enzymes post‐SAH (Table 1), which were illustrated briefly in Figure 2. These results suggest that changes in H_2_S production and H_2_S‐producing enzymes are spatiotemporal dependent, which is conceivable given that these enzymes are unevenly distributed in the brain. This differential expression potentially mediates the different effects of H_2_S in different brain regions. Therefore, further experiments are needed to explore the temporal and spatial distribution differences in H_2_S‐producing enzymes following SAH onset to better understand the underlying mechanism of H_2_S in this process.

**TABLE 1:** Changes of endogenous hydrogen sulfide (H_2_S) and H_2_S‐producing enzymes in the central nervous system after subarachnoid hemorrhage

Author	Time	Animal	Model	Tissue	Main results
Cui et al.^74^	2015	Rat	Prechiasmatic cistern single injection model	Brain tissue (PFC)	The CBS and 3‐MST protein level and endogenous production of H_2_S in the brain decreased at 4 days after SAH. Treatment with NaHS restored H_2_S production and the expressions of CBS and 3‐MST
Li et al.^84^	2017	Rat	SAH model using double blood injection into cisterna magna	PFC	The mRNA and protein level of CBS decreased in the PFC at 48 h after SAH, and H_2_S production slightly decreased, but with no significance
Han et al.^115^	2020	Human	SAH	CSF	The CBS, DAO, and 3‐MST protein levels increased within 48 h of SAH, which were significantly associated with increased IL‐6 level at 48 h in CSF and poor outcomes at 6 months after SAH onset
		Rat	SAH	CSF; parietal cortex and hippocampus	The expression of CBS, DAO, and 3‐MST increased first and then decreased in parietal cortex and hippocampus after SAH. Strong correlations between the increases in CBS, 3‐MST, and IL‐6 were detected
Duan et al.^87^	2020	Rat	SAH model via intravascular puncture method	Hippocampus	The concentration of H_2_S and CBS protein level increased at 24 h after SAH

Abbreviations: 3‐MST, 3‐mercaptopyruvate sulfurtransferase; CBS, cystathionine‐β‐synthetase; CSF, cerebrospinal fluid; DAO, d‐amino‐acid oxidase; IL‐6, interleukin‐6; PFC, prefrontal cortex; SAH, subarachnoid hemorrhage.

## 11 PROTECTIVE EFFECTS AND UNDERLYING MECHANISMS OF H_2_S IN SAH

Many studies have reported the neuroprotective roles of H_2_S in in vitro and in vivo models of SAH, as detailed in Table 2.

**TABLE 2:** The biological effects of hydrogen sulfide on subarachnoid hemorrhage

Author	Time	Animal/cell	Model	H_2_S administration	Main results
Cui et al.^74^	2015	Rat	SAH model via prechiasmatic cistern single injection	Intraperitoneal injection of sodium hydrosulfide (NaHS) (1.4 mg/kg; 5.6 mg/kg) at 4 h after SAH, qd, for 3 days	Exogenous NaHS treatment attenuate brain edema, blood–brain barrier disruption, brain cell apoptosis, inflammatory response, and cerebral vasospasm after SAH by elevating H_2_S production
		Primary rat cortical neurons; human umbilical vein endothelial cells	10‐μM OxyHb treatment	NaHS (15/30 μM)	Exogenous NaHS treatment protects neurons and endothelial function by antioxidant and antiapoptosis
Emmez et al.^108^	2016	Rat	Experimental cerebral vasospasm model of SAH induced by autologous arterial blood injection into cisterna magna	Intraperitoneal injection of a single dose of NaHS (0.18 mmol/kg) immediately after SAH	NaHS treatment reduced vasospasm at 24 h following SAH, which was correlated with the CSE expression in basilar artery
Cao et al.^73^	2016	Rat	SAH model via endovascular perforation method	Intraperitoneal injection of NaHS (5 mg/kg) at 30 min before SAH induction	Exogenous NaHS treatment attenuated brain edema formation and apoptotic cell death and improved neurological dysfunction at 24 h after SAH
Li et al.^83^	2016	Rat	SAH model using Suzuki's double blood injection with modification	Intraperitoneal injection of NaHS with a dose of 5.6 mg/kg at 2, 6, 24, and 46 h after SAH	NaHS treatment improved brain edema and neurobehavioral function, alleviated cognitive dysfunction, and attenuated neuronal cell death in the prefrontal cortex via Akt/ERK‐related antiapoptosis pathway and upregulating BDNF‐CREB expression
Li et al.^84^	2017	Rat	SAH model using double blood injection into cisterna magna	Lateral cerebral ventricle injection of 30 µl of 100‐mM l‐cysteine solution 30 min after SAH	l‐cysteine treatment inhibited cell apoptosis, upregulated CREB–BDNF expression, and promoted synaptic structure via the CBS/H_2_S pathway, ameliorated brain edema, improved neurobehavioral function
Shi et al.^85^	2017	Rat	SAH model via endovascular perforation method	Intraperitoneal injection of NaHS (1.4 mg/kg; 5.6 mg/kg) at 1 h after SAH	Exogenous NaHS treatment ameliorates neuronal apoptosis after SAH via the ROS‐MST1 pathway, alleviated brain edema and improved neurological function
Xiong et al.^75^	2020	Rat	SAH model using an endovascular perforation technique	Lateral cerebral ventricle injection of 30‐μl l‐cysteine solution at 100 mM 30 min after SAH	Exogenous l‐cysteine treatment can play a neuroprotective role by regulating neuroinflammation, complement deposition, oxidative stress, and endoplasmic reticulum stress
Duan et al.^79^	2020	Rat	SAH model via endovascular perforation technique	Intraperitoneal injection of NaHS (5.6 mg/kg), qd, for 4 days	Exogenous NaHS treatment reduced the cognitive impairment of rats after SAH by ameliorating neuroinflammation in microglia, potentially via the TLR4/NF‐κB pathway
Duan et al.^87^	2020	Rat	SAH model via intravascular puncture method	Intraperitoneal injection of NaHS at 14 μmol/kg, qd, for a week	Exogenous NaHS treatment plays an important neurological protective effect by modulating the function of the L‐type calcium channel and inhibiting apoptosis

Abbreviations: Akt, advanced protein kinase B; BDNF, brain‐derived neurotrophic factor; CBS, cystathionine‐β‐synthetase; CREB, cyclic adenosine monophosphate response element‐binding protein; CSE, cystathionine‐γ‐lyase; ERK, extracellular signal‐regulated kinase; MST1, mammalian sterile 20‐like kinase 1; NaHS, sodium hydrosulfide; NF‐κB, nuclear translocation of nuclear factor kappa BOxyHb, oxyhemoglobin; ROS, reactive oxygen species; SAH, subarachnoid hemorrhage; TLR4, toll‐like receptor 4.

### 11.1 Antiinflammatory effects

Neuroinflammation is an important mechanism of early brain injury after SAH and plays a central role in the development of post‐SAH complications.^68^, ^69^, ^70^ On SAH onset, erythrocytes entering the subarachnoid space are degraded to release hemoglobin, which is cleaved to produce free heme.^71^ Heme activates a nuclear factor kappa beta (NF‐κB)‐mediated proinflammatory cascade in microglia, releasing inflammatory factors such as tumor necrosis factor‐α (TNF‐α) and interleukin‐1β (IL‐1β).^72^ There is evidence that the appropriate administration of exogenous H_2_S can reduce neuroinflammation in rats after SAH. In 2016, researchers confirmed that the activation of glia cells and the secretion of proinflammatory cytokines, such as IL‐1β, IL‐10, IL‐18, and TNF‐α, were alleviated in SAH rats via intraperitoneal injection of NaHS.^73^, ^74^ Similarly, another study found that injection of exogenous l‐cysteine in the lateral ventricle inhibited microglial activation in the prefrontal cortex (PFC) and reduced the mRNA levels of IL‐1β and CD86.^75^ Toll‐like receptor 4 (TLR4) is widely expressed on the surface of glial cells, such as microglia, astrocytes, and oligodendrocytes in the CNS, and it plays an important role in the inflammatory response once activated by hemoglobin and its derivatives after SAH.^76^, ^77^, ^78^ Duan et al.^79^ reported that NaHS treatment potentially alleviates neuroinflammation in microglia via the TLR4/NF‐κB pathway. To summarize, there is evidence that H_2_S alleviates neuroinflammation after SAH, but there are few reports on the underlying mechanism, and more in‐depth studies should be conducted.

### 11.2 Antiapoptosis

Apoptosis is a highly regulated cell death process, including exogenous and intrinsic pathways, both of which eventually cause the cleaving of caspase‐3, which is the specific executor of apoptosis.^80^ Both pathways are regulated by B‐cell lymphoma 2 (Bcl‐2) protein families, such as Bcl‐2 and Bcl‐2 associated X (Bax); Bcl‐2 is antiapoptotic, whereas Bax is proapoptotic. Bcl‐2, Bax, and caspase‐3 are commonly used markers for detecting apoptosis.^81^

Apoptosis is one of the main consequences of early brain injury and is initiated within minutes to 24 h after SAH.^82^ Many studies have confirmed the antiapoptotic effect of H_2_S after SAH. Recent studies have shown that treatment with NaHS induced a significant reduction in the percentage of transferase‐mediated dUTP nick end labeling (Tunel)‐positive cells in the inferior basal temporal lobe tissue and the endothelium of the anterior cerebral artery (ACA) and middle cerebral artery (MCA) of rats compared with the SAH group.^74^ Similarly, NaHS pretreatment dramatically decreased the number of Tunel‐positive cells and the protein expression of cleaved caspase‐3 in SAH rats. Furthermore, high‐dose NaHS treatment was found to have a greater antiapoptotic effect than low‐dose NaHS treatment.^73^ Exogenous NaHS significantly attenuated neuronal cell death in the PFC, which was associated with a decrease in the Bax/Bcl‐2 ratio and suppression of caspase‐3 activation in rats 48 h after SAH. The authors hypothesized that this was achieved through the advanced protein kinase B/extracellular signal‐regulated kinase (Akt/ERK)‐related antiapoptosis pathway, as NaHS partially reversed the decreasing levels of p‐ERK and p‐Akt after SAH.^83^ The same team also reported that l‐cysteine treatment attenuated neuronal cell death in the PFC at 48 h after SAH. l‐cysteine is another donor of H_2_S, just as NaSH, further confirming the antiapoptotic effect of H_2_S.^84^ In other research, the underlying mechanisms of H_2_S in antiapoptosis were thought to be executed through inhibition of the activity of mammalian sterile 20‐like kinase 1 (MST1) protein. MST1 is a key serine‐threonine kinase that plays an important role in apoptotic cell death. Previous studies have shown that oxidative stress activates and cleaves MST1 to produce highly active cleaved MST1 (cl‐MST1).^85^ Subsequently, cl‐MST1 is translocated to the nucleus to induce neuronal apoptosis.^86^ Exogenous NaHS decreased the protein level of cl‐MST1 while increasing the full‐length MST1 expression; however, this could be reversed by chelerythrine, which could activate MST1 via caspase‐dependent cleavage.^85^ A study by Duan et al. provided another explanation of H_2_S in antiapoptosis. We observed that the expression of Bax and caspase‐3 was elevated, whereas Bcl‐2 protein level decreased in the SAH group; this was reversed in the SAH + NaHS and SAH + Bay K8644 (L‐type calcium channel opener) groups. Compared with the SAH + NaHS group, the expression of proapoptotic proteins was higher in the SAH + NaHS + nifedipine (a calcium channel agonist) group. Therefore, the authors concluded that the antiapoptotic effect of NaHS was partially weakened by nifedipine, indicating that the beneficial effect of H_2_S might be correlated with the L‐type calcium channel.^87^ This antiapoptotic effect has also been verified in vitro. Cultured primary rat cortical neurons (PCNs) and human umbilical vein endothelial cells (HUVECs) were exposed to OxyHb at a concentration of 10 μM for 24 h to establish the SAH model in vitro. Researchers examined the expression of active caspase‐3, and the results showed that both high and low doses of NaSH dose‐dependently decreased the expression of active caspase‐3 compared with the SAH group.^74^ The antiapoptotic effects of H_2_S reported in the above studies seem to agree with this finding. However, it is still not clear which of these antiapoptotic mechanisms play a dominant role and whether they cooperate or interfere with each other, and further studies are still necessary.

### 11.3 Antioxidation

Oxyhemoglobin and its metabolites are deemed major sources of reactive oxygen species (ROS) during the pathophysiology of SAH.^88^, ^89^ After the occurrence of SAH, the release of oxygenated hemoglobin, and mitochondrial dysfunction and overexpression of peroxidase, leads to excessive production of oxidative products that exceed the body's antioxidant capacity. These processes further oxidize cell lipids, proteins, and deoxyribonucleic acid (DNA), resulting in programmed cell death.^90^, ^91^ ROS can also activate TLR/NF‐κB/Mitogen‐activated protein kinase (MAPK), Kelch‐like ECH‐associated protein 1‐nuclear factor erythroid 2‐related factor 2‐antioxidant response elements (KEAP1–NRF2–ARE), eicosanoid, and other signaling pathways, and Nod‐like receptor protein 3 (NLRP3) inflammasomes to mediate inflammatory responses. The inflammatory response and oxidative stress promote and complement each other, leading to the adverse outcome of SAH.^69^

The antioxidation effect of H_2_S in SAH has been confirmed recently. There is evidence that the levels of ROS and malondialdehyde (MDA) were significantly elevated, and that glutathione peroxidase (GSH‐Px) and superoxide dismutase (SOD) were decreased in the brains of SAH rats, which were partially reversed by NaSH treatment.^74^ Furthermore, NaSH alleviated the increased ROS level induced by OxyHb in PCNs and HUVECs^74^; this was again verified in research by Shi et al.^85^ In a recent article, Dihydroethidium (DHE) assays indicated that the administration of l‐cysteine markedly reduced the ROS content in the brains of SAH rats.^75^ Nuclear factor erythroid 2‐related factor (NRF2) is recognized as a cellular protective factor, regulating the expression of genes encoding antioxidant, antiinflammatory, and detoxifying proteins. Heme oxygenase‐1 (HO‐1) promotes the removal of toxic heme and the production of biliverdin, iron ions, and carbon monoxide. HO‐1 and its products play a protective role against oxidative injury, regulating apoptosis, modulating inflammation, and promoting angiogenesis.^92^ Immunohistochemistry has confirmed increased levels of NRF2 and HO‐1 in the SAH group and further upregulation after l‐Cysteine administration.^75^ Taken together, these results suggested the antioxidant role of H_2_S. However, exact mechanisms are still unclear, with most studies focusing on phenotypes.

### 11.4 Reducing brain edema

Severe cerebral edema is considered an independent risk factor for the prognosis of patients with SAH.^93^ Cerebral edema is caused by the accumulation of extravascular fluid and metabolic disturbance of water and ions.^94^ Cerebral edema after SAH is considered to result from vasogenic cerebral edema caused by the destruction of the BBB permeability, and cytotoxic edema and ionic edema caused by abnormal water channels and ion channels on the cell membrane.^20^, ^95^, ^96^, ^97^, ^98^, ^99^ There is evidence that BBB permeability significantly increases in the acute stage after SAH.^100^, ^101^ The breakdown of the BBB may be attributed to the degradation of tight junction proteins (TJPs), which is mediated by matrix metalloproteinases (MMPs).^102^ As the main aquaporin in the CNS, aquaporin protein4 (AQP4) is mainly expressed in the terminal feet of astrocytes around blood vessels and is involved in the formation of cell edema.^103^ There is evidence that microglia activation and secretion of proinflammatory cytokines lead to AQP4 disorder and promote the formation of cerebral edema.^94^ Ionic edema occurs immediately after cytotoxic edema; driven by a cytotoxic edema‐caused ion gradient between the vascular compartment and interstitial fluid (ISF), ionic edema further aggravates cytotoxic edema.^104^

It has been reported that H_2_S attenuates brain edema formation partially by inhibiting the degradation of TJPs (including ZO‐1, occludin, and claudin‐5) by reducing MMP‐9 expression/activity and suppressing AQP4 expression on astrocytes by alleviating glial activation and proinflammatory cytokine secretion.^73^ Importantly, this was verified in another study that suggested that NaHS supplementation eases hyperhomocysteinemia‐induced BBB permeability and brain edema by inhibiting the mRNA expression and activity of MMP‐9.^105^ In addition, some other studies have reported that H_2_S could attenuate the damage of SAH to BBB integrity and the ensuing brain edema, as shown by the significant reduction in Evans blue extravasation and/or the mean value of brain water content in the brains of SAH rats by exogenous NaSH/l‐cysteine treatment. However, the underlying mechanisms were not illuminated in these articles.^74^, ^75^, ^84^

### 11.5 Anticerebral vasospasm

Cerebral vasospasm after aneurysmal SAH (aSAH) is defined as large and medium intracranial artery stenosis. Cerebral vasospasm can lead to local cerebral hypoperfusion and DCI and is one of the major causes of death and disability in patients with aSAH.^106^ The exact mechanism of vasospasm after SAH is not fully understood, but many mechanisms have been proposed, such as endothelial injury and microthrombi formation; smooth muscle contraction, resulting from the lysis of subarachnoid blood clots and blood degradation products and hemoglobin released into the subarachnoid space; decreased NO production leading to prolonged vasoconstriction; increased production and release of the potent vasoconstrictor endothelin‐1; cortical spreading depolarization; inflammation‐mediated oxidative stress and free radical damage to smooth muscle cells; and upregulation of apoptotic pathways.^12^, ^107^

Many studies have reported the role of H_2_S in cerebral vasospasm after SAH. One found a significant hemadostenosis of ACA and MCA in the SAH group compared with the sham groups. Remarkably, there was a significant difference in the cross‐sectional areas of ACA and MCA between the SAH + NaHS and SAH groups, indicating that vasospasm was alleviated by H_2_S.^74^ Another study also reported the alleviating effect of H_2_S on cerebral vasospasm.^108^ It was found that compared with the control and SAH groups, CSE and CBS enzyme expressions were both higher in the NaSH groups in the brain stem and basilar artery, especially CSE in the basilar artery. In the SAH group, the basilar artery luminal diameter (LD) values were decreased and the wall thickness (WT) values were increased, which was inversed in both the NaHS and SAH + NaHS groups. NaHS treatment significantly reduced vasospasm at 24 h following SAH and showed a vasodilatory effect on the basilar artery in both normal and SAH rats’ brains. The authors then employed two inhibitors, propargylglycine (PPG) and aminooxyacetic acid (AOAA), to reduce the expression of CSE and CBS, respectively. It was found that PPG and AOAA treatments exerted a vasoconstrictive effect, while NaSH treatment exerted a vasodilative effect in the basilar artery. Hence, the vasodilative effect of NaSH was correlated with CBS and CSE expression in the basilar artery, especially CSE. H_2_S‐mediated dilatation of middle cerebral arteries is partly mediated by the inhibition of L‐type Ca^2+^ channels, with an additional contribution by K^+^ channels.^109^, ^110^ A study using cranial windows in newborn pigs in 2019 revealed that sulforaphane (SFN) increased cerebral blood flow via activation of CSE/CBS‐catalyzed H_2_S formation in neurovascular cells followed by H_2_S‐induced activation of K_ATP_ and Ca^2+^‐ and voltage‐gated K channels of large conductance (BK channels) in arteriolar smooth muscle.^111^ Although the mechanism of vasospasm after SAH is not well understood, evidence of vasodilation by H_2_S has been reported. Nevertheless, more attention should be paid to this area to better manage and prevent this complication.

### 11.6 Additional mechanisms

In addition to all of the above, H_2_S plays a neuroprotective role through other mechanisms. As a well‐known neuroprotective factor, BDNF mRNA and protein expression have been found to be significantly decreased at 48 h after SAH compared with the sham group, which was reversed by NaHS/l‐cysteine treatment.^83^, ^84^ Besides, NaHS/l‐cysteine treatment also significantly reversed the decrease in phosphorylation of CREB protein in PFC. Phosphorylated CREB regulates the transcription of several genes, which was correlated with molecules involved in neuronal plasticity.^83^, ^84^ Under transmission electron microscopy, the number of degenerative synapses increased while the normal synapses decreased after SAH. Moreover, l‐cysteine treatment dramatically alleviated synaptic damage and upregulated the number of normal synapses in the SAH rats. Similarly, the decreasing synaptophysin (presynaptic marker) and increasing PSD95 (postsynaptic marker) in the PFC at 48 h post‐SAH were also reversed by l‐cysteine treatment.^84^ Furthermore, bath application of H_2_S donor NaHS promotes surface insertion of a‐amino‐3‐hydroxy‐5‐methylisoxazole‐4‐propionic acid receptor (AMPAR) via phosphorylation of GluR1 at serine‐831 and serine‐845 sites, which depends on a sulfhydration‐mediated mechanism.^112^ AMPAR mediates the majority of fast excitatory synaptic transmission and its dynamic regulation is one of the key elements that allow the nervous system to adapt to environment stimulations. Taken together, this suggests that exogenous H_2_S can regulate synaptic function and reduce synaptic injury. Furthermore, there is evidence that l‐cysteine treatment could significantly reduce complement deposition and ER damage.^75^ Indeed, l‐cysteine has been shown to reduce the expression of the complement factors, C1q, C3α, and its receptor C3aR1, and the deposition of C1q in the PFC in SAH rats. Glucose‐related protein 78 (GRP78) is a marker for ER stress, while C/EBP homologous protein (CHOP) leads to cell death at the end stage of ER stress. l‐cysteine has been shown to reduce CHOP and GRP78 levels, with the number of CHOP‐ and GRP78‐positive cells shown to decrease in the rat after SAH according to the results of western blot assays and immunohistochemistry. Unfortunately, the authors did not further elucidate the underlying mechanisms of these effects, which require further efforts in future.

## 12 CLINICAL STUDY OF H_2_S IN SAH

Clinical studies on H_2_S in SAH are rarely reported. One study demonstrated that patients with the gain‐of‐function 844 WT/ins genotypes were less likely to experience DCI relative to those with the 844 WT/WT genotype, while those with the decrease‐in‐function genotype 1080 TT was more likely to experience DCI relative to those with 1080 CC and CT genotypes. However, serum homocysteine levels did not correlate with the extent of either angiographic vasospasm or DCI. Hence, it was concluded that H_2_S may mediate protection from DCI following aSAH through a mechanism that is independent of macrovascular vasodilation.^113^ A few years later, another study reported that the insertion allele of the 844ins68 CBS insertion polymorphism was independently associated with aSAH, while the GG genotype of rs234706 was associated with an unfavorable outcome both at discharge and last follow‐up.^114^ While the insertion allele of 844ins68 has been linked to a gain‐of‐function of the CBS enzyme, others have reported that this polymorphism had no effect on plasma homocysteine levels. Increased CBS activity may exert its neuroprotective effects through the alteration of H_2_S levels, independent of clinical vasospasm and DCI. However, the two prospective observational studies are insufficient to explain the role of H_2_S in SAH. To better understand this issue, larger sample size interventional clinical studies are needed in the coming years.

## 13 CONCLUSIONS

**FIGURE 3:**
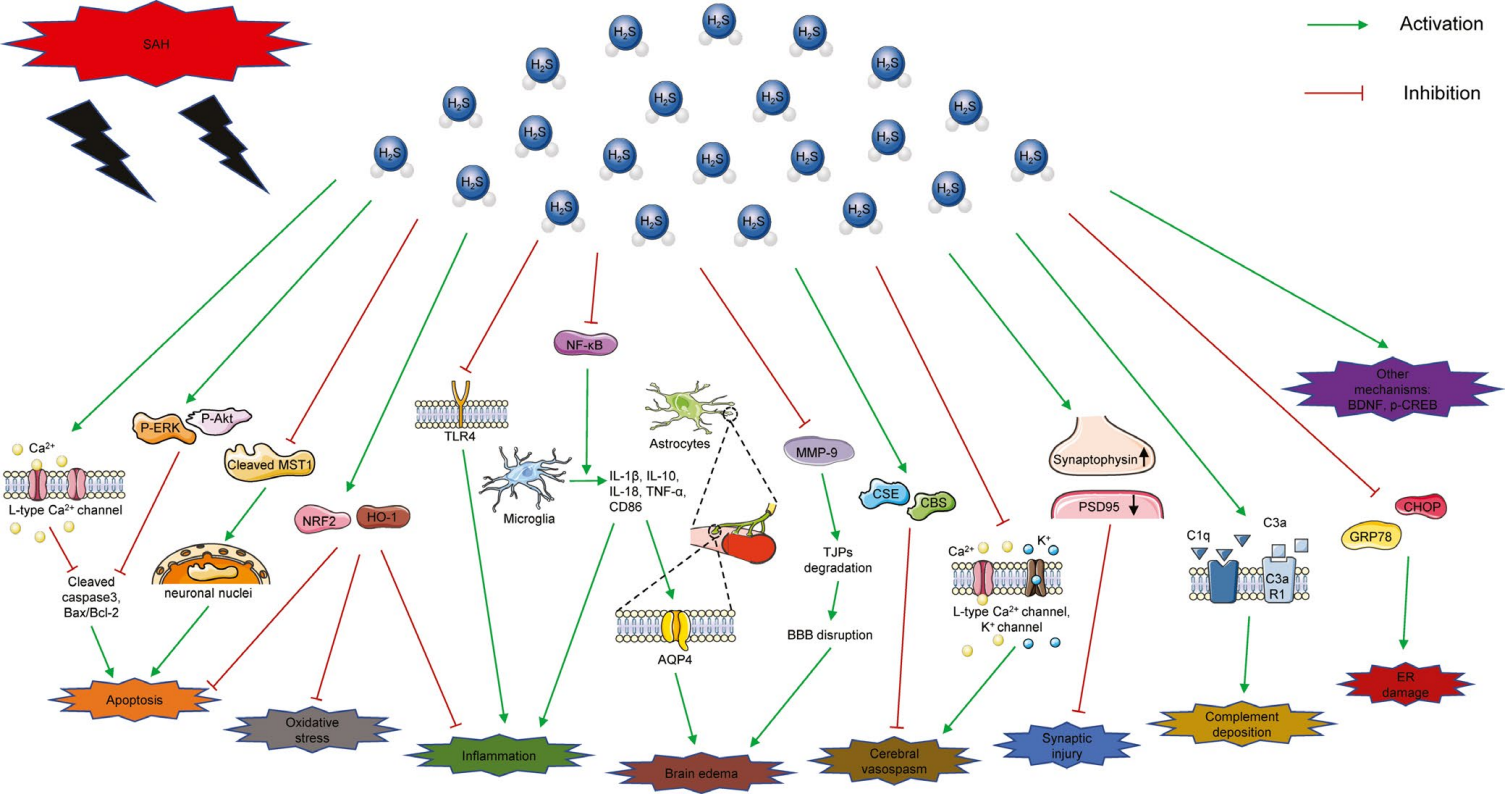
Potential therapeutic effects and mechanisms of H_2_S in SAH. This figure illustrates the pathophysiological mechanism of brain injury induced by SAH and the possible roles of H_2_S in it. AQP4, aquaporin protein4; Bax, Bcl‐2‐associated X; BBB, blood–brain barrier; Bcl‐2, B‐cell lymphoma 2; BDNF, brain‐derived neurotrophic factor; C3aR1, C3a receptor 1; CBS, cystathionine‐β‐synthetase; CHOP, C/EBP homologous protein; CSE, cystathionine‐γ‐lyase; ER, endoplasmic reticulum; GRP78, glucose‐related protein 78; HO‐1, heme oxygenase‐1; IL‐1β, interleukin‐1β; MMP‐9, metalloproteinases‐9; MST, mercaptopyruvate sulfurtransferase; NF‐κB, nuclear factor kappa B; NRF2, nuclear factor erythroid 2‐related factor 2; p‐Akt, phosphorylated advanced protein kinase B; p‐CREB, phosphorylated cyclic adenosine monophosphate response element‐binding protein; p‐ERK, phosphorylated extracellular signal‐regulated kinase; PSD95, postsynaptic density 95; TJP, tight junction proteins; TLR, toll‐like receptor; TNF‐α, tumor necrosis factor‐α

In summary, many studies have demonstrated the neuroprotection of H_2_S in SAH. Although the specific mechanism has not been fully elucidated, it is potentially related to antiinflammatory, antiapoptosis, and antioxidation effects, and alleviating cerebral edema and vasospasm (Figure 3). However, the pathophysiology of SAH is complicated, and more studies are needed to further understand the role of H_2_S in SAH. Since the production of H_2_S after SAH is spatiotemporal dependent, it is supposed to explore the changes of H_2_S level and its producing enzymes in different regions, such as cortex, hippocampus, cerebellum, and cerebrovascular tissue. In addition, CBS, 3‐MST, or other enzymes can be overexpressed or knockdown by gene regulation or transgenic technology, and changes in inflammation, apoptosis, oxidative stress, and other related pathway proteins can be detected by RNA sequence or proteomics technology, so as to clarify the specific role of H_2_S in different brain regions and its potential mechanism. Moreover, H_2_S has a neuroprotective effect only in limited doses and can be toxic in excess. Therefore, extensive research is needed to determine safe delivery methods and optimal dosages of H_2_S. When the time is right, clinical studies should also be carried out widely. To our knowledge, there are few clinical studies on H_2_S therapy up to now. In conclusion, we believe that H_2_S therapy will become a viable alternative for SAH treatment in the near future.

## CONFLICTS OF INTEREST

The authors declare that they have no competing interests.

## AUTHOR CONTRIBUTIONS

DFL and LLW reviewed the design and wrote the manuscript; GJL and SXW drafted the manuscript; YW and YW revised the manuscript; JW and XOS involved in critical editing of the manuscript. All the authors read and approved the final version of the manuscript for publication.

## Data Availability

Data sharing not applicable to this article as no datasets were generated or analyzed during the current study.
